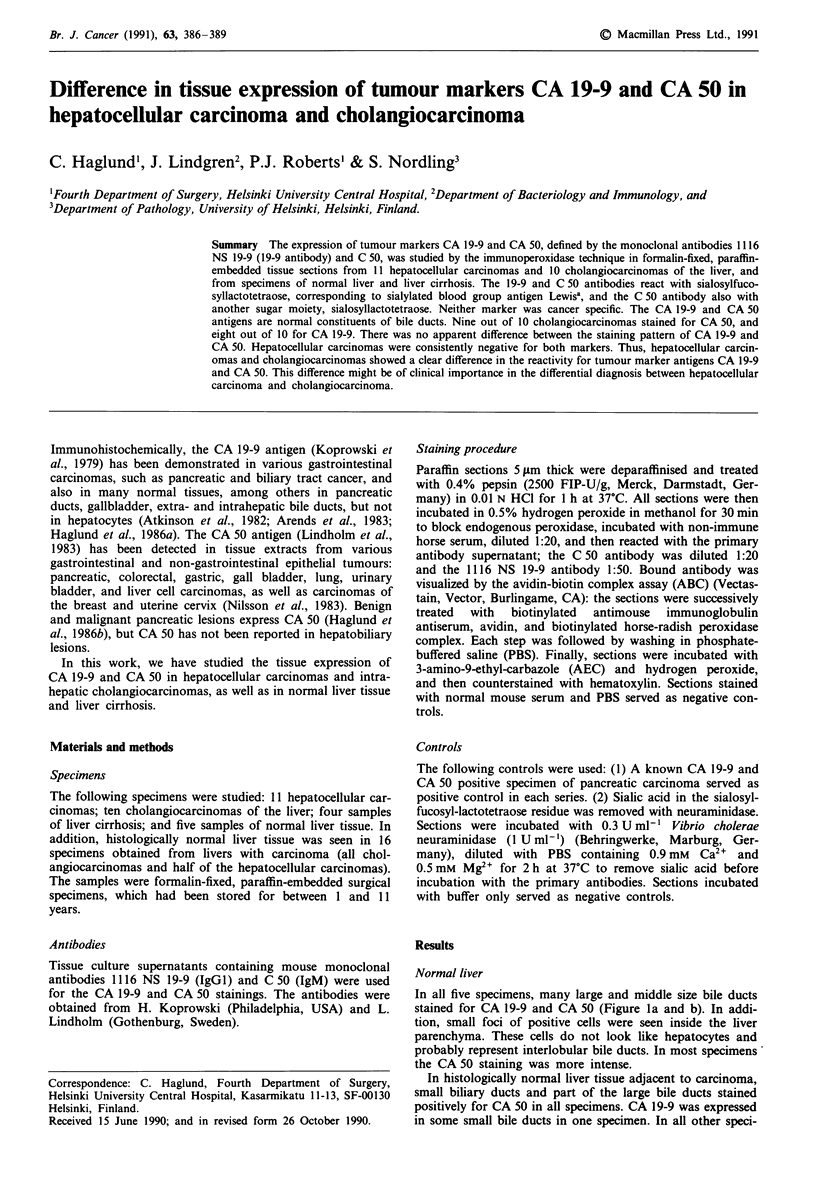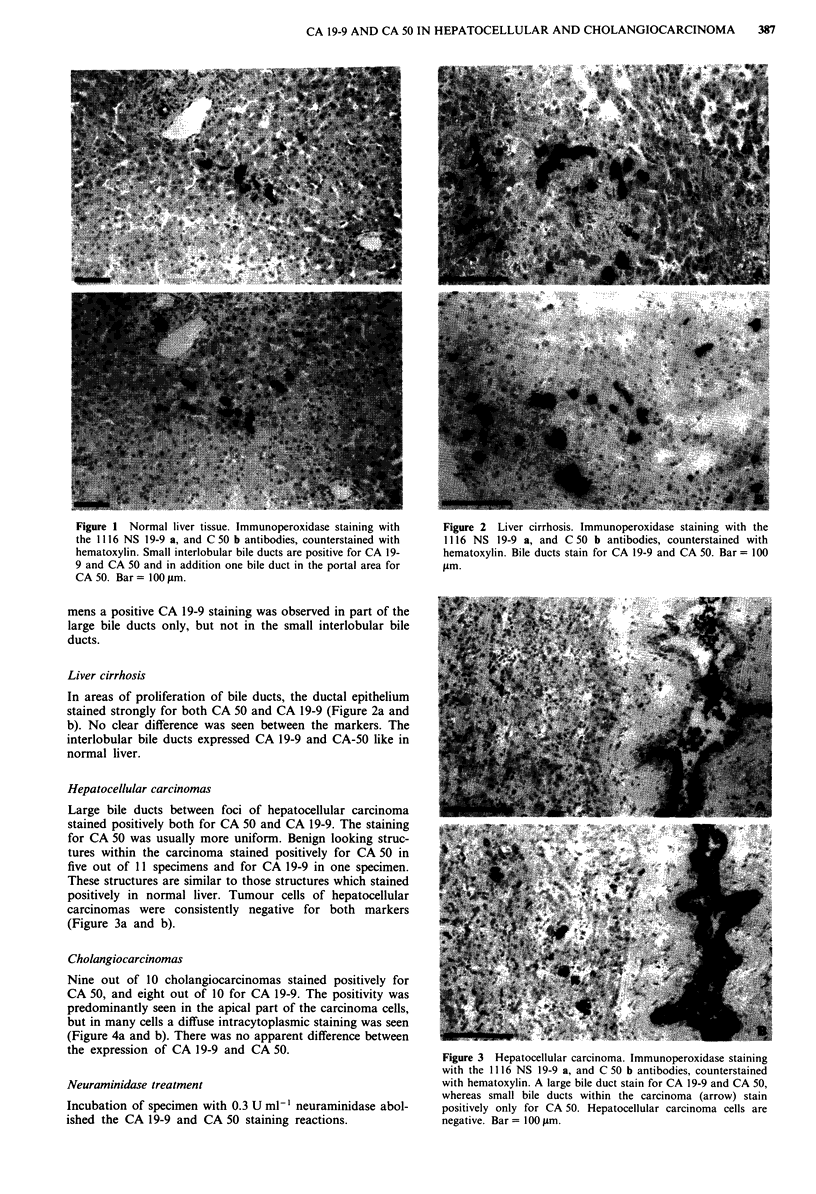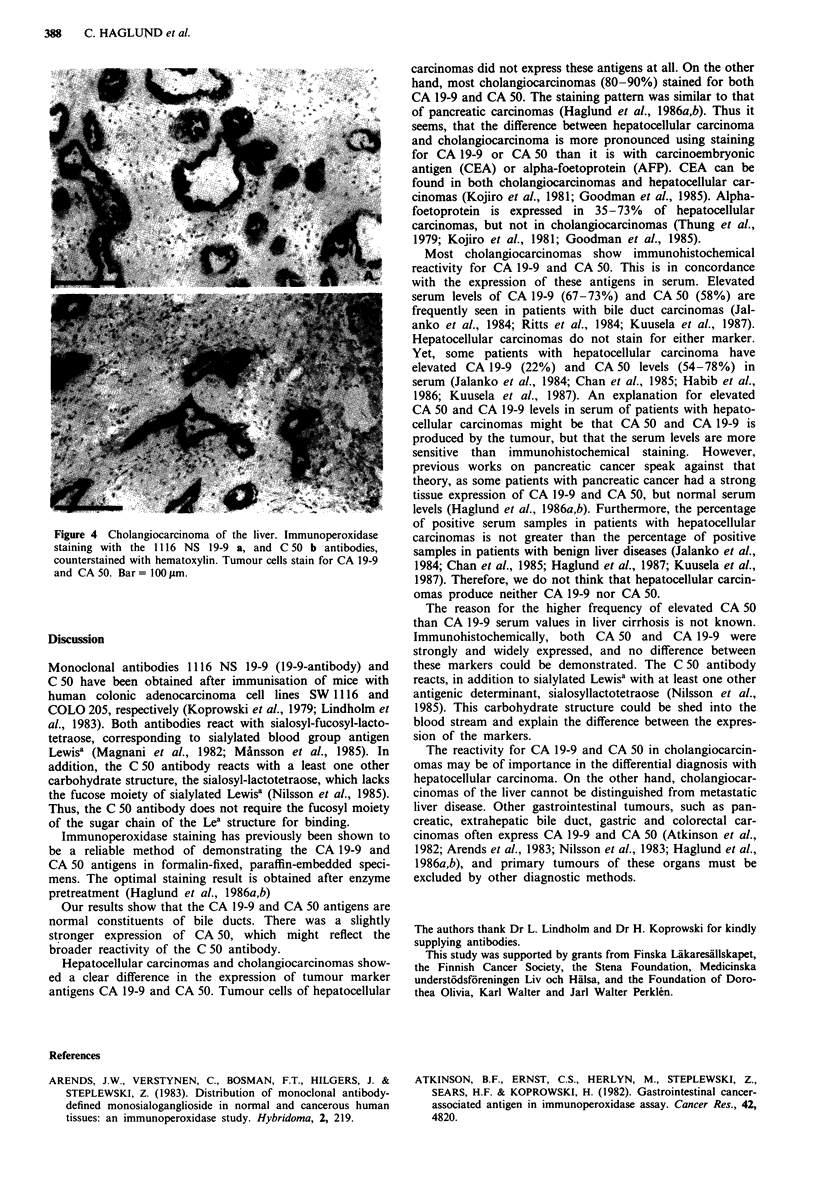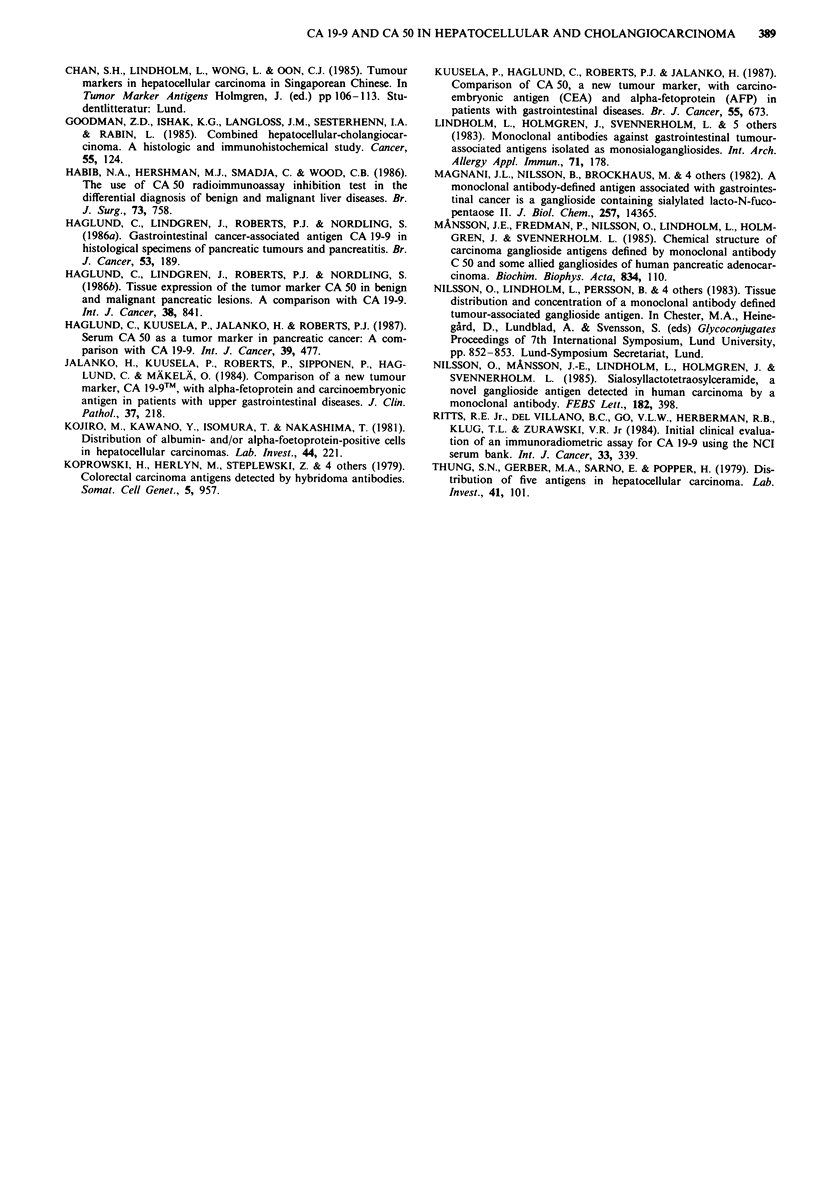# Difference in tissue expression of tumour markers CA 19-9 and CA 50 in hepatocellular carcinoma and cholangiocarcinoma.

**DOI:** 10.1038/bjc.1991.90

**Published:** 1991-03

**Authors:** C. Haglund, J. Lindgren, P. J. Roberts, S. Nordling

**Affiliations:** Fourth Department of Surgery, Helsinki University Central Hospital, Finland.

## Abstract

**Images:**


Br. J. Cancer (1991), 63, 386-389 © Macmillan Press Ltd., 1991

### Difference in tissue expression of tumour markers CA 19-9 and CA 50 in hepatocellular carcinoma and cholangiocarcinoma

C. Haglund[1], J. Lindgren[2], P.J. Roberts[1] & S. Nordling[3]

[1]*Fourth Department of Surgery, Helsinki University Central Hospital,* [2]*Department of Bacteriology and Immunology, and* [3]*Department of Pathology, University of Helsinki, Helsinki, Finland.*

**Summary** The expression of tumour markers CA 19-9 and CA 50, defined by the monoclonal antibodies 1116 NS 19-9 (19-9 antibody) and C 50, was studied by the immunoperoxidase technique in formalin-fixed, paraffin-embedded tissue sections from 11 hepatocellular carcinomas and 10 cholangiocarcinomas of the liver, and from specimens of normal liver and liver cirrhosis. The 19-9 and C 50 antibodies react with sialosylfucosyllactotetraose, corresponding to sialylated blood group antigen Lewis[a], and the C 50 antibody also with another sugar moiety, sialosyllactotetraose. Neither marker was cancer specific. The CA 19-9 and CA 50 antigens are normal constituents of bile ducts. Nine out of 10 cholangiocarcinomas stained for CA 50, and eight out of 10 for CA 19-9. There was no apparent difference between the staining pattern of CA 19-9 and CA 50. Hepatocellular carcinomas were consistently negative for both markers. Thus, hepatocellular carcinomas and cholangiocarcinomas showed a clear difference in the reactivity for tumour marker antigens CA 19-9 and CA 50. This difference might be of clinical importance in the differential diagnosis between hepatocellular carcinoma and cholangiocarcinoma.

Immunohistochemically, the CA 19-9 antigen (Koprowski *et al.*, 1979) has been demonstrated in various gastrointestinal carcinomas, such as pancreatic and biliary tract cancer, and also in many normal tissues, among others in pancreatic ducts, gallbladder, extra- and intrahepatic bile ducts, but not in hepatocytes (Atkinson *et al.*, 1982; Arends *et al.*, 1983; Haglund *et al.*, 1986*a*). The CA 50 antigen (Lindholm *et al.*, 1983) has been detected in tissue extracts from various gastrointestinal and non-gastrointestinal epithelial tumours: pancreatic, colorectal, gastric, gall bladder, lung, urinary bladder, and liver cell carcinomas, as well as carcinomas of the breast and uterine cervix (Nilsson *et al.*, 1983). Benign and malignant pancreatic lesions express CA 50 (Haglund *et al.*, 1986*b*), but CA 50 has not been reported in hepatobiliary lesions.

In this work, we have studied the tissue expression of CA 19-9 and CA 50 in hepatocellular carcinomas and intrahepatic cholangiocarcinomas, as well as in normal liver tissue and liver cirrhosis.

### Materials and methods

### *Specimens*

The following specimens were studied: 11 hepatocellular carcinomas; ten cholangiocarcinomas of the liver; four samples of liver cirrhosis; and five samples of normal liver tissue. In addition, histologically normal liver tissue was seen in 16 specimens obtained from livers with carcinoma (all cholangiocarcinomas and half of the hepatocellular carcinomas). The samples were formalin-fixed, paraffin-embedded surgical specimens, which had been stored for between 1 and 11 years.

### *Antibodies*

Tissue culture supernatants containing mouse monoclonal antibodies 1116 NS 19-9 (IgG1) and C 50 (IgM) were used for the CA 19-9 and CA 50 stainings. The antibodies were obtained from H. Koprowski (Philadelphia, USA) and L. Lindholm (Gothenburg, Sweden).

Correspondence: C. Haglund, Fourth Department of Surgery, Helsinki University Central Hospital, Kasarmikatu 11-13, SF-00130 Helsinki, Finland.
Received 15 June 1990; and in revised form 26 October 1990.

### *Staining procedure*

Paraffin sections 5 $\mu$m thick were deparaffinised and treated with 0.4% pepsin (2500 FIP-U/g, Merck, Darmstadt, Germany) in 0.01 N HCl for 1 h at 37°C. All sections were then incubated in 0.5% hydrogen peroxide in methanol for 30 min to block endogenous peroxidase, incubated with non-immune horse serum, diluted 1:20, and then reacted with the primary antibody supernatant; the C 50 antibody was diluted 1:20 and the 1116 NS 19-9 antibody 1:50. Bound antibody was visualized by the avidin-biotin complex assay (ABC) (Vectastain, Vector, Burlingame, CA): the sections were successively treated with biotinylated antimouse immunoglobulin antiserum, avidin, and biotinylated horse-radish peroxidase complex. Each step was followed by washing in phosphate-buffered saline (PBS). Finally, sections were incubated with 3-amino-9-ethyl-carbazole (AEC) and hydrogen peroxide, and then counterstained with hematoxylin. Sections stained with normal mouse serum and PBS served as negative controls.

### *Controls*

The following controls were used: (1) A known CA 19-9 and CA 50 positive specimen of pancreatic carcinoma served as positive control in each series. (2) Sialic acid in the sialosylfucosyl-lactotetraose residue was removed with neuraminidase. Sections were incubated with 0.3 U ml$^{-1}$ *Vibrio cholerae* neuraminidase (1 U ml$^{-1}$) (Behringwerke, Marburg, Germany), diluted with PBS containing 0.9 mM $Ca^{2+}$ and 0.5 mM $Mg^{2+}$ for 2 h at 37°C to remove sialic acid before incubation with the primary antibodies. Sections incubated with buffer only served as negative controls.

### Results

### *Normal liver*

In all five specimens, many large and middle size bile ducts stained for CA 19-9 and CA 50 (Figure 1a and b). In addition, small foci of positive cells were seen inside the liver parenchyma. These cells do not look like hepatocytes and probably represent interlobular bile ducts. In most specimens the CA 50 staining was more intense.

In histologically normal liver tissue adjacent to carcinoma, small biliary ducts and part of the large bile ducts stained positively for CA 50 in all specimens. CA 19-9 was expressed in some small bile ducts in one specimen. In all other speci-

**Figure 1** Normal liver tissue. Immunoperoxidase staining with the 1116 NS 19-9 **a**, and C 50 **b** antibodies, counterstained with hematoxylin. Small interlobular bile ducts are positive for CA 19-9 and CA 50 and in addition one bile duct in the portal area for CA 50. Bar = 100 μm.

**Figure 2** Liver cirrhosis. Immunoperoxidase staining with the 1116 NS 19-9 **a**, and C 50 **b** antibodies, counterstained with hematoxylin. Bile ducts stain for CA 19-9 and CA 50. Bar = 100 μm.

mens a positive CA 19-9 staining was observed in part of the large bile ducts only, but not in the small interlobular bile ducts.

*Liver cirrhosis*

In areas of proliferation of bile ducts, the ductal epithelium stained strongly for both CA 50 and CA 19-9 (Figure 2a and b). No clear difference was seen between the markers. The interlobular bile ducts expressed CA 19-9 and CA-50 like in normal liver.

*Hepatocellular carcinomas*

Large bile ducts between foci of hepatocellular carcinoma stained positively both for CA 50 and CA 19-9. The staining for CA 50 was usually more uniform. Benign looking structures within the carcinoma stained positively for CA 50 in five out of 11 specimens and for CA 19-9 in one specimen. These structures are similar to those structures which stained positively in normal liver. Tumour cells of hepatocellular carcinomas were consistently negative for both markers (Figure 3a and b).

*Cholangiocarcinomas*

Nine out of 10 cholangiocarcinomas stained positively for CA 50, and eight out of 10 for CA 19-9. The positivity was predominantly seen in the apical part of the carcinoma cells, but in many cells a diffuse intracytoplasmic staining was seen (Figure 4a and b). There was no apparent difference between the expression of CA 19-9 and CA 50.

*Neuraminidase treatment*

Incubation of specimen with 0.3 U $ml^{-1}$ neuraminidase abolished the CA 19-9 and CA 50 staining reactions.

**Figure 3** Hepatocellular carcinoma. Immunoperoxidase staining with the 1116 NS 19-9 **a**, and C 50 **b** antibodies, counterstained with hematoxylin. A large bile duct stain for CA 19-9 and CA 50, whereas small bile ducts within the carcinoma (arrow) stain positively only for CA 50. Hepatocellular carcinoma cells are negative. Bar = 100 μm.

**Figure 4** Cholangiocarcinoma of the liver. Immunoperoxidase staining with the 1116 NS 19-9 **a**, and C 50 **b** antibodies, counterstained with hematoxylin. Tumour cells stain for CA 19-9 and CA 50. Bar = 100 μm.

### Discussion

Monoclonal antibodies 1116 NS 19-9 (19-9-antibody) and C 50 have been obtained after immunisation of mice with human colonic adenocarcinoma cell lines SW 1116 and COLO 205, respectively (Koprowski *et al.*, 1979; Lindholm *et al.*, 1983). Both antibodies react with sialosyl-fucosyl-lacto-tetraose, corresponding to sialylated blood group antigen Lewis[a] (Magnani *et al.*, 1982; Månsson *et al.*, 1985). In addition, the C 50 antibody reacts with a least one other carbohydrate structure, the sialosyl-lactotetraose, which lacks the fucose moiety of sialylated Lewis[a] (Nilsson *et al.*, 1985). Thus, the C 50 antibody does not require the fucosyl moiety of the sugar chain of the Le[a] structure for binding.

Immunoperoxidase staining has previously been shown to be a reliable method of demonstrating the CA 19-9 and CA 50 antigens in formalin-fixed, paraffin-embedded specimens. The optimal staining result is obtained after enzyme pretreatment (Haglund *et al.*, 1986*a*,*b*)

Our results show that the CA 19-9 and CA 50 antigens are normal constituents of bile ducts. There was a slightly stronger expression of CA 50, which might reflect the broader reactivity of the C 50 antibody.

Hepatocellular carcinomas and cholangiocarcinomas showed a clear difference in the expression of tumour marker antigens CA 19-9 and CA 50. Tumour cells of hepatocellular carcinomas did not express these antigens at all. On the other hand, most cholangiocarcinomas (80–90%) stained for both CA 19-9 and CA 50. The staining pattern was similar to that of pancreatic carcinomas (Haglund *et al.*, 1986*a*,*b*). Thus it seems, that the difference between hepatocellular carcinoma and cholangiocarcinoma is more pronounced using staining for CA 19-9 or CA 50 than it is with carcinoembryonic antigen (CEA) or alpha-foetoprotein (AFP). CEA can be found in both cholangiocarcinomas and hepatocellular carcinomas (Kojiro *et al.*, 1981; Goodman *et al.*, 1985). Alpha-foetoprotein is expressed in 35–73% of hepatocellular carcinomas, but not in cholangiocarcinomas (Thung *et al.*, 1979; Kojiro *et al.*, 1981; Goodman *et al.*, 1985).

Most cholangiocarcinomas show immunohistochemical reactivity for CA 19-9 and CA 50. This is in concordance with the expression of these antigens in serum. Elevated serum levels of CA 19-9 (67–73%) and CA 50 (58%) are frequently seen in patients with bile duct carcinomas (Jalanko *et al.*, 1984; Ritts *et al.*, 1984; Kuusela *et al.*, 1987). Hepatocellular carcinomas do not stain for either marker. Yet, some patients with hepatocellular carcinoma have elevated CA 19-9 (22%) and CA 50 levels (54–78%) in serum (Jalanko *et al.*, 1984; Chan *et al.*, 1985; Habib *et al.*, 1986; Kuusela *et al.*, 1987). An explanation for elevated CA 50 and CA 19-9 levels in serum of patients with hepatocellular carcinomas might be that CA 50 and CA 19-9 is produced by the tumour, but that the serum levels are more sensitive than immunohistochemical staining. However, previous works on pancreatic cancer speak against that theory, as some patients with pancreatic cancer had a strong tissue expression of CA 19-9 and CA 50, but normal serum levels (Haglund *et al.*, 1986*a*,*b*). Furthermore, the percentage of positive serum samples in patients with hepatocellular carcinomas is not greater than the percentage of positive samples in patients with benign liver diseases (Jalanko *et al.*, 1984; Chan *et al.*, 1985; Haglund *et al.*, 1987; Kuusela *et al.*, 1987). Therefore, we do not think that hepatocellular carcinomas produce neither CA 19-9 nor CA 50.

The reason for the higher frequency of elevated CA 50 than CA 19-9 serum values in liver cirrhosis is not known. Immunohistochemically, both CA 50 and CA 19-9 were strongly and widely expressed, and no difference between these markers could be demonstrated. The C 50 antibody reacts, in addition to sialylated Lewis[a] with at least one other antigenic determinant, sialosyllactotetraose (Nilsson *et al.*, 1985). This carbohydrate structure could be shed into the blood stream and explain the difference between the expression of the markers.

The reactivity for CA 19-9 and CA 50 in cholangiocarcinomas may be of importance in the differential diagnosis with hepatocellular carcinoma. On the other hand, cholangiocarcinomas of the liver cannot be distinguished from metastatic liver disease. Other gastrointestinal tumours, such as pancreatic, extrahepatic bile duct, gastric and colorectal carcinomas often express CA 19-9 and CA 50 (Atkinson *et al.*, 1982; Arends *et al.*, 1983; Nilsson *et al.*, 1983; Haglund *et al.*, 1986*a*,*b*), and primary tumours of these organs must be excluded by other diagnostic methods.

The authors thank Dr L. Lindholm and Dr H. Koprowski for kindly supplying antibodies.

This study was supported by grants from Finska Läkaresällskapet, the Finnish Cancer Society, the Stena Foundation, Medicinska understödsföreningen Liv och Hälsa, and the Foundation of Dorothea Olivia, Karl Walter and Jarl Walter Perklén.

### References

ARENDS, J.W., VERSTYNEN, C., BOSMAN, F.T., HILGERS, J. & STEPLEWSKI, Z. (1983). Distribution of monoclonal antibody-defined monosialoganglioside in normal and cancerous human tissues: an immunoperoxidase study. *Hybridoma*, **2**, 219.

ATKINSON, B.F., ERNST, C.S., HERLYN, M., STEPLEWSKI, Z., SEARS, H.F. & KOPROWSKI, H. (1982). Gastrointestinal cancer-associated antigen in immunoperoxidase assay. *Cancer Res.*, **42**, 4820.

CHAN, S.H., LINDHOLM, L., WONG, L. & OON, C.J. (1985). Tumour markers in hepatocellular carcinoma in Singaporean Chinese. In *Tumor Marker Antigens* Holmgren, J. (ed.) pp 106–113. Studentlitteratur: Lund.

GOODMAN, Z.D., ISHAK, K.G., LANGLOSS, J.M., SESTERHENN, I.A. & RABIN, L. (1985). Combined hepatocellular-cholangiocarcinoma. A histologic and immunohistochemical study. *Cancer*, **55**, 124.

HABIB, N.A., HERSHMAN, M.J., SMADJA, C. & WOOD, C.B. (1986). The use of CA 50 radioimmunoassay inhibition test in the differential diagnosis of benign and malignant liver diseases. *Br. J. Surg.*, **73**, 758.

HAGLUND, C., LINDGREN, J., ROBERTS, P.J. & NORDLING, S. (1986*a*). Gastrointestinal cancer-associated antigen CA 19-9 in histological specimens of pancreatic tumours and pancreatitis. *Br. J. Cancer*, **53**, 189.

HAGLUND, C., LINDGREN, J., ROBERTS, P.J. & NORDLING, S. (1986*b*). Tissue expression of the tumor marker CA 50 in benign and malignant pancreatic lesions. A comparison with CA 19-9. *Int. J. Cancer*, **38**, 841.

HAGLUND, C., KUUSELA, P., JALANKO, H. & ROBERTS, P.J. (1987). Serum CA 50 as a tumor marker in pancreatic cancer: A comparison with CA 19-9. *Int. J. Cancer*, **39**, 477.

JALANKO, H., KUUSELA, P., ROBERTS, P., SIPPONEN, P., HAGLUND, C. & MÄKELÄ, O. (1984). Comparison of a new tumour marker, CA 19-9™, with alpha-fetoprotein and carcinoembryonic antigen in patients with upper gastrointestinal diseases. *J. Clin. Pathol.*, **37**, 218.

KOJIRO, M., KAWANO, Y., ISOMURA, T. & NAKASHIMA, T. (1981). Distribution of albumin- and/or alpha-foetoprotein-positive cells in hepatocellular carcinomas. *Lab. Invest.*, **44**, 221.

KOPROWSKI, H., HERLYN, M., STEPLEWSKI, Z. & 4 others (1979). Colorectal carcinoma antigens detected by hybridoma antibodies. *Somat. Cell Genet.*, **5**, 957.

KUUSELA, P., HAGLUND, C., ROBERTS, P.J. & JALANKO, H. (1987). Comparison of CA 50, a new tumour marker, with carcinoembryonic antigen (CEA) and alpha-fetoprotein (AFP) in patients with gastrointestinal diseases. *Br. J. Cancer*, **55**, 673.

LINDHOLM, L., HOLMGREN, J., SVENNERHOLM, L. & 5 others (1983). Monoclonal antibodies against gastrointestinal tumour-associated antigens isolated as monosialogangliosides. *Int. Arch. Allergy Appl. Immun.*, **71**, 178.

MAGNANI, J.L., NILSSON, B., BROCKHAUS, M. & 4 others (1982). A monoclonal antibody-defined antigen associated with gastrointestinal cancer is a ganglioside containing sialylated lacto-N-fucopentaose II. *J. Biol. Chem.*, **257**, 14365.

MÅNSSON, J.E., FREDMAN, P., NILSSON, O., LINDHOLM, L., HOLMGREN, J. & SVENNERHOLM. L. (1985). Chemical structure of carcinoma ganglioside antigens defined by monoclonal antibody C 50 and some allied gangliosides of human pancreatic adenocarcinoma. *Biochim. Biophys. Acta*, **834**, 110.

NILSSON, O., LINDHOLM, L., PERSSON, B. & 4 others (1983). Tissue distribution and concentration of a monoclonal antibody defined tumour-associated ganglioside antigen. In Chester, M.A., Heinegård, D., Lundblad, A. & Svensson, S. (eds) *Glycoconjugates* Proceedings of 7th International Symposium, Lund University, pp. 852–853. Lund-Symposium Secretariat, Lund.

NILSSON, O., MÅNSSON, J.-E., LINDHOLM, L., HOLMGREN, J. & SVENNERHOLM. L. (1985). Sialosyllactotetraosylceramide, a novel ganglioside antigen detected in human carcinoma by a monoclonal antibody. *FEBS Lett.*, **182**, 398.

RITTS, R.E. Jr., DEL VILLANO, B.C., GO, V.L.W., HERBERMAN, R.B., KLUG, T.L. & ZURAWSKI, V.R. Jr (1984). Initial clinical evaluation of an immunoradiometric assay for CA 19-9 using the NCI serum bank. *Int. J. Cancer*, **33**, 339.

THUNG, S.N., GERBER, M.A., SARNO, E. & POPPER, H. (1979). Distribution of five antigens in hepatocellular carcinoma. *Lab. Invest.*, **41**, 101.